# Southwest Harvest for Health: An Adapted Mentored Vegetable Gardening Intervention for Cancer Survivors

**DOI:** 10.3390/nu13072319

**Published:** 2021-07-06

**Authors:** Cindy K. Blair, Prajakta Adsul, Dolores D. Guest, Andrew L. Sussman, Linda S. Cook, Elizabeth M. Harding, Joseph Rodman, Dorothy Duff, Ellen Burgess, Karen Quezada, Ursa Brown-Glaberman, Towela V. King, Erika Baca, Zoneddy Dayao, Vernon Shane Pankratz, Sally Davis, Wendy Demark-Wahnefried

**Affiliations:** 1 Department of Internal Medicine, University of New Mexico, MSC07-4025, Albuquerque, NM 87131, USA; padsul@salud.unm.edu (P.A.); dguest@salud.unm.edu (D.D.G.); lcook@salud.unm.edu (L.S.C.); ubrown-glaberman@salud.unm.edu (U.B.-G.); zdayao@salud.unm.edu (Z.D.); vpankratz@salud.unm.edu (V.S.P.); 2 University of New Mexico Comprehensive Cancer Center, Albuquerque, NM 87102, USA; asussman@salud.unm.edu (A.L.S.); jrodman@salud.unm.edu (J.R.); emburgess@salud.unm.edu (E.B.); kaquezada@salud.unm.edu (K.Q.); 3 Department of Family and Community Medicine, University of New Mexico, Albuquerque, NM 87131, USA; 4 Department of Rehabilitation and Movement Science, University of Vermont, Burlington, VT 05405, USA; elizabeth.harding@med.uvm.edu; 5 Albuquerque Area Extension Master Gardener Program, NMSU Cooperative Extension Service, Albuquerque, NM 87107, USA; duffsdales@gmail.com; 6 School of Medicine, University of New Mexico, Albuquerque, NM 87131, USA; tvking@salud.unm.edu (T.V.K.); eabaca@salud.unm.edu (E.B.); 7 Department of Pediatrics, University of New Mexico, Albuquerque, NM 87131, USA; sdavis@salud.unm.edu; 8 University of New Mexico Prevention Research Center, Albuquerque, NM 87131, USA; 9 Department of Nutrition Sciences, University of Alabama at Birmingham, Birmingham, AL 35294, USA; demark@uab.edu; 10 O’Neal Comprehensive Cancer Center at the University of Alabama at Birmingham, Birmingham, AL 35294, USA

**Keywords:** cancer survivors, gardening, vegetable, horticultural therapy, quality of life

## Abstract

Harvest for Health is a home-based vegetable gardening intervention that pairs cancer survivors with Master Gardeners from the Cooperative Extension System. Initially developed and tested in Alabama, the program was adapted for the different climate, growing conditions, and population in New Mexico. This paper chronicles the feasibility, acceptability, and preliminary efficacy of “Southwest Harvest for Health”. During the nine-month single-arm trial, 30 cancer survivor-Master Gardener dyads worked together to establish and maintain three seasonal gardens. Primary outcomes were accrual, retention, and satisfaction. Secondary outcomes were vegetable and fruit (V and F) intake, physical activity, and quality of life. Recruitment was diverse and robust, with 30 survivors of various cancers, aged 50–83, roughly one-third minority, and two-thirds females enrolled in just 60 days. Despite challenges due to the COVID-19 pandemic, retention to the nine-month study was 100%, 93% reported “good-to-excellent” satisfaction, and 87% “would do it again.” A median increase of 1.2 servings of V and F/day was documented. The adapted home-based vegetable gardening program was feasible, well-received, and resulted in increased V and F consumption among adult cancer survivors. Future studies are needed to evaluate the effectiveness of this program and to inform strategies to increase the successful implementation and further dissemination of this intervention.

## 1. Introduction

In 2019, there were 16.9 million cancer survivors living in the United States (U.S.) [1]. This number is expected to increase to 22.1 million by the year 2030 due to the growth and aging of the population [1,2] and could be substantially higher with further improvements in screening rates, access to care, and effective treatments. Due to the tremendous improvements in early detection and treatment, 56% of cancer survivors are living 10 years or more beyond their diagnosis [1]. However, many cancer survivors are at increased risk for treatment-related comorbidities, including cardiovascular disease, diabetes, and reduced quality of life [3,4,5,6,7,8,9]. This has led to preventive health being an important aspect of cancer survivorship [10]. Adherence to a healthy lifestyle has been recommended to improve health outcomes and quality of life and for reduce cancer recurrence and premature mortality [11]. 

Guidelines for cancer survivorship provide recommendations for adherence to a healthy lifestyle for individuals “living with, through, and beyond cancer” [11,12]. These guidelines encourage cancer survivors to achieve and maintain a healthy lifestyle through weight management, eating a diet low in red meats, sugars, and refined grains, and high in whole grains and vegetables and fruit (V and F), and engaging in regular physical activity. Non-adherence to these recommendations has been associated with increased risk of second malignancies, diabetes, cardiovascular disease, disability, and premature mortality [13,14,15]. Although several interventions have proven efficacious in improving diet and physical activity for cancer survivors [16,17,18], very few have been successfully adapted for different populations or settings, which limits their potential to impact population health [18,19,20]. 

Harvest for Health is a home-based vegetable gardening intervention that pairs cancer survivors with certified Master Gardeners from the Cooperative Extension System (Extension). Extension is a program within the U.S. Department of Agriculture that operates through the education and outreach arm of land-grant universities nationwide [21]. The Master Gardener Program [22], one of Extension’s many education and outreach programs, provides research-based education and training in horticulture to U.S. residents nationwide. Upon completion of their training, the certified Master Gardeners educate and serve their local communities through various projects. The Harvest for Health program has tremendous potential for sustainability, and widespread dissemination since Master Gardener programs exist in all states and territories of the U.S. and typically require 50–100 h of volunteer service annually to maintain certification [21,22,23]. The intervention, developed and initially tested in Alabama by Demark-Wahnefried and colleagues, has resulted in increased vegetable consumption and leisure-time physical activity and improvements in health-related quality of life (HRQOL) and physical functioning in cancer survivors [24,25,26].

Widespread adoption and implementation of evidence-based interventions are critical for achieving population-level impact. To achieve widespread implementation, evidence-based interventions need to be adapted to different populations and contexts. We adapted Harvest for Health for the different climate, growing conditions, and population of New Mexico (NM), which was reported previously [27]. We then pilot tested the adapted intervention, Southwest Harvest for Health, and examined the feasibility, acceptability, and preliminary efficacy. Our primary objective is to determine the feasibility and acceptability of the mentored gardening intervention by assessing recruitment, retention, and adherence rates, monitoring adverse events, and evaluating satisfaction with the program in a different population and context. The secondary objective is to explore changes in V and F intake, physical activity, and HRQOL. 

## 2. Methods

A detailed description of the study protocol was published previously [27]. Briefly, this was a single-arm pilot study whereby all participants received the nine-month mentored vegetable gardening intervention. The study was conducted from February 2020 through November 2020. The baseline assessment preceded the COVID-19 pandemic; the six- and nine-month follow-up assessments both occurred during the COVID-19 pandemic. The University of New Mexico (UNM) Health Sciences Center Institutional Review Board approved this study. Written informed consent was obtained from all participants prior to the baseline assessment.

### 2.1. Study Participants

The targeted sample size for this pilot study was 30 adult cancer survivors. Oncologists and nurse navigators referred cancer survivors to the study by giving them a study flyer. Additionally, recruitment flyers were distributed via cancer survivor groups, community centers, and other community locations. Interested individuals contacted study staff by email or telephone and were screened for eligibility. Eligible individuals were then scheduled for their baseline assessment.

Eligibility included residence in Bernalillo or Sandoval counties (together comprising most of the Albuquerque-area population). Adults aged 50 years and older with a diagnosis of any type of cancer were eligible. Patients with metastatic cancer were eligible with physician approval. Additional eligibility criteria included: (1) resided in a location that could accommodate a 1.2 m × 2.4 m raised bed garden or four (62.2 cm × 52.1 cm) garden containers, and have access to outdoor running water; (2) able to speak, read, and understand English; and (3) able to participate in the 9-month intervention. Exclusion criteria included: (1) any medical condition that substantially limited activities of daily living (e.g., bending, stooping, walking) that would preclude gardening; (2) eating more than five daily servings of V and F; (3) spending more than 150 min per week in moderate-to-vigorous intensity physical activity; and (4) recent experience (within the past year) with vegetable gardening. 

### 2.2. Harvest for Health Gardening Intervention

A detailed description of the Southwest Harvest for Health intervention has been previously published [27]. Similar to the original Harvest for Health study developed in Alabama [24,25,26,28], the current pilot study is a community-based, mutually beneficial partnership between UNM and the New Mexico State University Extension Master Gardener Program [29,30,31]. Harvest for Health pairs each cancer survivor with a certified Master Gardener from Extension [24,25,26,28]. Together, the participant/Master Gardener dyads work to establish and maintain three seasonal gardens at the participants’ homes. Participants receive gardening supplies, plants and seeds, and print materials (study notebook). The study notebook includes articles on safety tips while gardening (e.g., arthritis, protecting hands and feet), instructions for assembling the garden boxes or raised beds, helpful gardening resources from Extension (e.g., “Home Vegetable Gardening in New Mexico” publication), and a planning guide for suggested crops to grow each season. However, most of the gardening knowledge is acquired by working with their Master Gardener mentor. Dyads are asked to communicate every two weeks throughout the intervention, alternating between home visits and telephone or email. The Master Gardener mentor provides information and support related to plants and care of the garden (care of the soil, insect/pest management, watering crops) and helps troubleshooting problems that develop (insects/pests, too little water, too much water or wind, slow growth, etc.). 

Due to statewide public health rules implemented during the COVID-19 pandemic, the following changes were made to the study design. The statewide stay-at-home order (March 2020) resulted in issues with scheduling the home deliveries of the larger gardening supplies. Instead, a “drive-through” distribution center was established, and members of the study team loaded the gardening supplies, plants, and seeds into the participants’ vehicles. All participants received four gardening containers (62.2 cm × 52.1 cm each; easier to transport than the larger raised bed kits) and a smaller selection of seedlings (limited access to/hours of nurseries and gardening stores). Monthly home visits by the Master Gardeners to their participants’ gardens were replaced with an extra telephone call or email, which occurred for the duration of the nine-month study. Participants were encouraged to send photos of their garden to their Master Gardener.

### 2.3. Primary Outcomes and Measures: Feasibility and Acceptability

The feasibility and acceptability of the home-based mentored vegetable gardening intervention were determined by achieving the following goals: (1) recruitment of 30 adult cancer survivors; (2) retention of ≥80% of the participants; (3) achievement of ≥80% adherence to the intervention; (4) absence of serious adverse events either attributable or possibly attributable to the gardening intervention; and (5) achievement of high acceptability/satisfaction rates with the intervention (≥75%). The cut-points for retention and adherence were selected a priori for comparison with the earlier Harvest for Health studies [25,26].

Retention was calculated as the percentage of participants who completed the post-intervention assessment. Intervention adherence was assessed by the number of completed monthly surveys on garden status, the number of monthly garden photos that were emailed or texted to the study team, and the self-reported frequency of communicating with their Master Gardener mentor (≥2 times per month was specified). Overall satisfaction with the program was assessed via a debriefing survey that was mailed to the study participants. Questions included the following: (1) “How would you rate your experience with the Southwest Harvest for Health study?” (6 response items ranging from excellent to very poor); (2) “Based on your experience, would you do it again?” (5 response items ranging from “yes, most definitely” to “no, not at all”); and (3) “How likely are you to recommend this program to someone else?” (5 response items ranging from “very likely” to “very unlikely”). Additional questions elicited the perceived effect of the intervention on V and F intake, physical activity, and psychosocial well-being, as well as intention to continue gardening on their own.

### 2.4. Secondary Outcomes and Measures: Health and Lifestyle Outcomes

The intervention health and lifestyle outcomes were assessed at baseline during a home visit. The six- and nine-month follow-up assessments were conducted via telephone and paper or digital surveys to accommodate pandemic restrictions. 

Daily V and F consumption was assessed using the “Eating at America’s Table Screener” (EATS) either in person (baseline visit; with food props) or via telephone (follow-up visits; with show cards mailed to participants). The EATS screener [32], developed by the National Cancer Institute, comprises 10 questions on frequency (ranging from never to multiple times per day) and quantity (ranging from none to more than two cups) for selected foods. The total number of servings of V and F (fresh, canned, frozen, or 100% juice) were calculated according to the screener scoring recommendations [33]. Questions related to the consumption of white potatoes, fried potatoes, beans and legumes, and mixed vegetable dishes were not included in the computation.

Device-based measures of physical activity and sedentary behavior were measured using an inclinometer/accelerometer. Participants were asked to wear the activPAL3, a small device attached mid-thigh, day and night for seven days at three time points: at the beginning, at six months, and at the end of the study. Participants recorded the following in their sleep diary: the time the device was attached, the time it was removed and reattached (if applicable), and the time they went to bed at night and woke up the next morning. The activPAL monitor provides accurate measures of sedentary time (sitting or lying), standing, and stepping [34,35,36,37]. The a priori outcomes of interest were changes in steps per day, time spent stepping at both light-intensity and moderate-intensity cadence, and time spent engaged in sedentary behavior. 

Self-reported physical activity was assessed using Godin’s Leisure-Time Physical Activity Questionnaire via telephone (after wearing the activPAL3 monitor). The Godin questionnaire assesses the amount of structured exercise (e.g., walking, sports) completed in sessions lasting ten minutes or longer in duration [38,39]. The frequency (times per week) and average duration (minutes) is recorded for types of exercise based on three levels of intensity: mild exercise (minimal effort, no perspiration; example: easy walking), moderate exercise (not exhausting, light perspiration; example: fast walking), and strenuous exercise (heart beats rapidly, sweating; example: jogging or running). Since this questionnaire only assesses structured exercise, it is not directly comparable to the number of steps or time spent stepping measured by a research-grade device. Self-reported Sedentary Behavior was assessed using the Sedentary Behavior Questionnaire (SBQ; paper survey completed after wearing the activPAL3 monitor). The SBQ survey assesses time spent in nine common activities, such as watching television, using a computer, reading, or doing artwork/crafts [40]. Frequency options for each type of activity include none, 15 min or less, 30 min, or 1, 2, 3, 4, or 5 h, or 6 h or more. Time spent in sedentary behavior is assessed for a typical weekday and for a typical weekend day.

HRQOL was measured using PROMIS (Patient-Reported Outcomes Measurement Information System) measures [41]. The 8-item short forms were used to assess domains in mental health (anxiety and depression), physical health (physical function, fatigue, pain, sleep disturbance, and sleep impairment), and social health (satisfaction with social roles and activities, i.e., social functioning). These instruments are valid and reliable for use in diverse clinical samples [42,43,44,45]. Surveys were scored using the free Health Measures Scoring Service (https://www.assessmentcenter.net/ac_scoringservice; accessed 3 July 2021). The service provides T-scores, which represent a linear transformation of the raw scores normed to the general population, with a mean of 50 and a standard deviation of 10. For physical function and social functioning, higher scores indicate better functioning; for the remaining domains, higher scores indicate worse functioning.

### 2.5. Other Outcomes and Measures

The Social Provisions Scale was used to assess participants’ perceived level of social support [46]. This survey includes six subscales: reassurance of worth (how other people recognize one’s value), social integration (sense of belonging), guidance (information/advice), nurturance (sense of being needed by others), attachment (emotional closeness), and reliable alliance (assurance that other people will provide assistance if needed). Scores on each item range from one (strongly disagree) to four (strongly agree), with subscale scores ranging from four to sixteen. Total perceived social support is the sum of the six subscales (range 24 to 96). Higher scores represent greater support.

### 2.6. Data Analysis

The primary outcomes of this pilot study were the accrual and retention of the cancer survivors and paired Master Gardeners throughout the nine-month intervention, as well as satisfaction with the program. Secondary outcomes included trends in V and F consumption, physical activity, and HRQOL. The processing of the activPAL data to calculate time spent engaged in physical activities and sedentary activities has been previously described [47]. All activPAL variables were standardized to a 15-h day to limit the effect of within and between-person variability in awake/wear time. Descriptive characteristics of the enrolled cancer survivors are presented as frequencies and percentages or medians with interquartile range (IQR). Similar to most pilot studies, our pilot study was not powered to detect significant nor clinically meaningful changes in measures of V and F intake, physical activity, and HRQOL. However, estimates of the pre-post changes will be useful in planning for a future larger trial. As diet and physical activity are seasonally influenced [18,19], the changes observed since baseline are of primary interest; however, change for both the mid- and post-intervention follow-up are included in the results. Data were summarized as medians and IQRs, and the 6- and 9-month differences were presented as medians and IQRs. The Wilcoxon Signed-Rank Test was used to evaluate the 6- and 9-month change. SAS (version 9.4) was used to perform the statistical analyses.

## 3. Results

### 3.1. Feasibility 

Of the 40 cancer survivors who were screened, 10 were ineligible, and the remaining 30 were enrolled in the pilot study. The top two reasons for ineligibility included current and successful experience with vegetable gardening and living outside of the study catchment area. Retention in this nine-month intervention was 100%. No adverse events were attributable or possibly attributable to the gardening intervention.

The characteristics of the 30 cancer survivors enrolled in this study are included in Table 1. The median age at study enrollment was 68 years (range 50 to 83 years). Most study participants were female (70%), non-Hispanic white (73%), and slightly over half had graduated from college. Eighty-four percent reported their health as good, very good, or excellent, while the median number of comorbidities reported was 3 (range 0 to 8). The median time since cancer diagnosis was 5 years (range 1 to 17 years). While a variety of cancer types were represented, the most common were breast, prostate, and lung.

### 3.2. Adherence

Adherence during the intervention was moderately high for completing the monthly gardening activity surveys. Eighty percent of participants completed all six surveys, and the remaining 20% completed five surveys. However, adherence was only modest for sending photos of the garden to the study team (average = 56%; range of 47% to 63%). Most participants (89%) reported communicating with their Master Gardener at least twice a month; on average, 72% reported three or more times per month. The remainder (11%) reported less frequent communication. On average, 40% and 50% of study participants reported working in their garden several times a day and once a day, respectively. The majority (60%) of participants reported working 15–29 min each time they worked in their garden. 

### 3.3. Acceptability

Upon completion of the study, 83% of participants responded “probably yes” or “yes, most definitely” to planning to continue the garden and plant on their own; 13% responded “maybe”, and 3% responded “probably no”. When asked if they planned to expand their garden, 69% responded “probably yes” or “yes, most definitely”, 14% responded “maybe”, and 17% responded “probably no”. However, among the latter two groups (31%), half of the participants had already expanded their garden during the study, based on the monthly surveys or photos of their garden. Most participants (90%) rated their experience with Southwest Harvest for Health as “very good” or “excellent” (3% “good”, 7% “fair”) and were “likely” or “very likely” to recommend the program to another cancer survivor (10% “neutral”). Eighty-seven percent responded “yes, most definitely” or “probably yes” that they would “do it again” based on their experience (the remaining 13% were divided between “maybe” and “probably no”).

### 3.4. Secondary Outcomes

Physical activity, V and F intake, and HRQOL scores are reported in Table 2. At study completion, the greatest improvement was observed for the number of servings per day of V and F. Compared to baseline, the median change at post-intervention follow-up was 1.2 additional servings per day. The median change in device-measured physical activity was a decrease of 478 steps per day that corresponded to 1.1 and 4.2 fewer minutes of stepping at a light-intensity and moderate-intensity, respectively. Sedentary behavior increased by 14.8 min per day. On average, there was no appreciable change in physical or mental quality of life; however, there was a modest improvement in social functioning (median: 3.3 points; IQR: -1.4, 10.9).

### 3.5. Other Outcomes 

There were no appreciable changes in any of the social support subscales for both the 6- and 9-month follow-up assessments (data not shown). For five of the six subscales at both time points, the median change was zero; for nurturance, the median change was one point (both time points).

**Figure 1:**
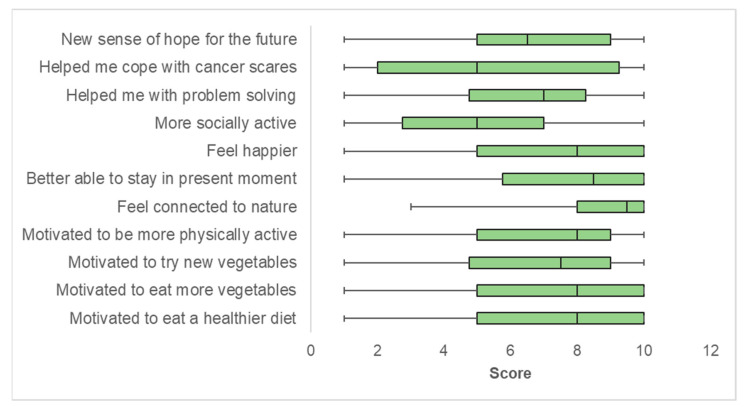
Impact of a home-based vegetable gardening intervention on diet, physical activity, and well-being. Shaded areas represent the interquartile range. Vertical lines within each shaded region represent the median score. Whiskers represent the minimum and maximum scores for the questions, ranging from 1 (not at all) to 10 (very much).

Overall, most participants reported that the gardening experience motivated them to eat a healthier diet, eat more vegetables, or try new vegetables (median scores of 7 or 8 out of 10; Figure 1). Most participants also reported motivation to be more physically active (median score of 8 out of 10); additional activities reported included yard work and walking, with a few participants reporting yoga and other exercises. There was also a positive impact of the gardening experience on well-being (Figure 1). Average scores were highest for feeling connected to nature when gardening and mindfulness, i.e., being better able to stay in the present moment (median scores of 9.5 and 8.5 out of 10, respectively). Average scores were lowest for being more socially active (likely due to COVID-19). Sixty percent of cancer survivors reported that gardening helped them cope with pain, anxiety about test results, either fear of or having received a diagnosis of cancer recurrence or a second cancer, or dealing with cancer in a family member.

## 4. Discussion

This study explored the feasibility and acceptability of a home-based, mentored vegetable gardening intervention adapted for middle-aged and older cancer survivors living in the Southwest U.S. It represents the first systematic adaptation of the original Harvest for Health program for a different state/region of the U.S. Despite being conducted entirely during the COVID-19 pandemic, which necessitated remote instead of in-person mentoring, the gardening intervention was feasible. This was demonstrated by the high recruitment and retention rates, as well as the moderately high adherence rates. Satisfaction with the intervention was also high, based on the percentage of cancer survivors rating their experience, being willing to “do it again”, and the likelihood of recommending the program to other cancer survivors.

The feasibility metrics of Southwest Harvest for Health compare favorably with the earlier pilot studies conducted in Alabama. The retention rates in this one-year (Alabama; AL) or nine-month (New Mexico; NM) vegetable gardening intervention have been very high (91–100%) [24,25,26], suggesting that the variety of gardening activities and benefits may help prevent satiation, which is more common with other lifestyle promotion programs. Satisfaction rates with Southwest Harvest for Health were also similar to those of the original Harvest for Health pilot programs: 93% vs. 100%, respectively, rating the experience as good to excellent; and 87% vs. 85–100%, respectively, stating they would “do it again” [24,25,26]. Another similarity between all these pilot studies is the expansion or plans to expand the garden space (NM: 84%; AL: 70–89%) as well as intention to continue vegetable gardening beyond the study (NM: 90%; AL: 85–100%) [24,25,26].

Although the current pilot study was a small, single-arm study, the health behavior outcomes compare favorably to the earlier pilot studies. We observed a meaningful overall increase of 1.2 servings per day of V and F, similar to the earlier pilot studies, which reported increases of 0.9 servings per day [25,26]. The magnitude of this change is particularly notable in view that increases of half a serving a day have been considered clinically important in previous work [48]. While maintaining a small, home vegetable garden likely represents light-intensity physical activities, these activities may serve as a gateway for additional physical activity. Similar to the earlier studies, participants in the current study reported being motivated by their garden to do more yard work and to walk more. In contrast to the earlier studies, but not surprisingly, few participants mentioned joining a fitness center during the pandemic. Engagement in these types of activities may have prevented more substantial declines in overall activity compared with other studies of adults during the COVID-19 pandemic [49]. While there are fewer reports of declines in HRQOL that have been systematically observed during COVID-19, Grajek et al. recently reported declines of 2% among 450 patients actively treated for cancer [50]. Therefore, the high satisfaction with the gardening intervention may have mitigated steeper reductions in HRQOL that may have been noted otherwise. 

As with many research studies conducted during 2020, the potential effects of COVID-19 on this home-based gardening intervention must be taken into consideration. While the baseline assessment was conducted prior to the declaration of COVID-19 as a pandemic, both the intervention and follow-up assessment were conducted during the pandemic. Thus, diet, physical activity, and HRQOL could also have been affected by stay-at-home orders and recommendations, social distancing, and closed fitness centers, parks, and swimming pools. Additionally, a large proportion of the study participants were at higher risk for Sars-CoV-2 infection and complications, and thus greatly limited their time away from home or interactions with other people. 

Limitations of this pilot study were the lack of a control group and the potential effect of seasonal variation on V and F intake and physical activity. Due to the colder winters in New Mexico (compared to Alabama), we shortened the one-year intervention to nine months. Some studies have shown that diet, especially consumption of fresh V and F, may be influenced by season [51,52]. Similarly, physical activities, especially outdoor activities, may be influenced by season [53,54,55]. In the Southwest US, we would expect more outdoor activity during the spring and fall seasons. 

## 5. Conclusions and Future Directions

The adapted home-based vegetable gardening intervention was feasible, safe, and well-received by middle-aged and older cancer survivors living in the Southwest U.S. Future directions include moving from efficacy trials to effectiveness/pragmatic trials to observe the impact of this promising intervention under real-world conditions. The Cooperative Extension System is ideally situated for delivering health promotion programs in community settings, which can greatly expand their reach to a broader and more diverse population. Further research is needed to optimize the implementation of Harvest for Health within the Extension Master Gardener Programs within a state and ideally to other states across the nation.

## Figures and Tables

**Table 1:** Baseline characteristics of the cancer survivors participating in Southwest Harvest for Health.

Characteristics	Median (IQR) or Frequency (%)
Age (range 50 to 83)	68 (64, 72)
Sex	
Female	21 (70%)
Male	9 (30%)
Race-ethnicity	
Non-Hispanic White	22 (73%)
Hispanic White	6 (20%)
Other	2 (7%)
Education	
No college degree	13 (43%)
College degree	17 (57%
Cancer type	
Breast	11 (37%)
Prostate	6 (20%)
Lung	4 (13%)
Other ^a^	9 (30%)
Treatment received ^b^	
Surgery	23 (77%)
Radiation	22 (73%)
Chemotherapy	10 (33%)
Hormone therapy	12 (40%)
Other	2 (7%)
Years since cancer diagnosis (range 1 to 17)	5 (2, 8)
Self-reported general health	
Excellent	2 (7%)
Very good	5 (17%)
Good	18 (60%)
Fair	5 (17%)
Poor	0 (0%)
Number of comorbidities (range 0 to 8)	3 (2, 4)
BMI (kg/m^2^)	28.8 (24.4, 32.1)

^a^ Colorectal, melanoma, endometrial, lymphoma, ovarian, Merkel cell carcinoma. ^b^ Percentages do not total 100%, since some participants may have had more than one type of treatment.

**Table 2:** Change in health-related outcomes during the mentored gardening study.

	Pre−Intervention (Pre-COVID−19) Median (IQR)	Mid-Intervention (during COVID-19) Median (IQR)	Post-Intervention (during COVID−19) Median (IQR)	Pre-Mid Median Difference (IQR) *p*-Value ^a^	Pre-Post Median Difference (IQR) *p*-Value ^a^
Lifestyle Behaviors					
V and F (servings per day)	3.8 (2.5, 6.3)	5.5 (3.6, 7.2)	5.3 (3.7, 6.3)	0.9 (−0.3, 2.2) *p* = 0.006	1.2 (−0.4, 2.2) *p* = 0.03
Physical activity ^a^					
Self−Report (Minutes per day)					
Light intensity	11.3 (2.1, 17.1)	4.3 (0, 25.7)	5.3 (0, 25.7)	−1.4 (−10, 4.3) *p* = 0.39	−0.7 (−10, 6.3) *p* = 0.66
Moderate intensity	0 (0, 7.1)	0 (0, 8.6)	0 (0, 12.9)	0 (0, 0) *p* = 0.28	0 (0, 2.9) *p* = 0.19
Device−based Measures					
Steps per day	6781 (5523, 8633)	6403 (4796, 7854)	5831 (4287, 8038)	−792 (−1631, 464) *p* = 0.02	−478 (−1832, 312) *p* = 0.05
Minutes per day:					
Standing	256.4 (201.5, 286.0)	248.1 (181.5, 324.1)	250.3 (180.6, 314.8)	0.5 (−26.8, 29.0) *p* = 0.82	5.8 (−44.3, 49.1) *p* = 0.49
Light intensity	39.6 (30.7, 51.2)	38.3 (29.2, 48.8)	37.3 (26.8, 47.6)	−0.1 (−5.8, 3.0) *p*−0.46	−1.1 (−10.6, 3.4) *p* = 0.20
Moderate intensity	49.5 (38.5, 70.6)	49.3 (36.9, 64.4)	45.8 (30.4, 64.3)	−6.1 (−15.1, 4.0) *p* = 0.01	−4.2 (−13.8, 3.7) *p* = 0.06
Sedentary behavior					
Self-Report (Minutes per day)	517.8 (379.2, 623.4)	469.2 (355.8, 591.6)	507.0 (379.2, 618.0)	−54.0 (−114.0, 6.0) *p* = 0.06	12.0 (−114.0, 90) *p* = 0.87
Device-based Measure (Minutes per day)	440.5 (361.4, 505.8)	457.2 (366.9, 529.0)	457.8 (405.2, 510.3)	27.7 (−40.8, 51.1) *p* = 0.50	14.8 (−35.8, 54.6) *p* = 0.63
HRQOL ^b^					
Physical					
Physical function	47.0 (42.9, 53.1)	44.8 (33.9, 52.4)	45.6 (39.3, 52.8)	−0.1 (−4.9, 2.0) *p* = 0.50	0.0 (−7.0, 4.3) *p* = 0.39
Fatigue	50.5 (45.6, 58.2)	50.7 (46.9, 59.2)	49.8 (43.0, 57.5)	1.1 (−2.1, 3.7) *p* = 0.41	−0.9 (−5.7, 1.5) *p* = 0.28
Pain	55.4 (40.7, 58.3)	54.4 (50.3, 57.5)	50.6 (40.7, 58.7)	0.0 (−2.4, 0.3) *p* = 0.57	0.0 (−7.1, 0.5) *p* = 0.09
Sleep disturbance	50.4 (48.7, 53.2)	51.3 (50.2, 53.5)	52.0 (49.3, 52.9)	−0.7 (−2.3, 3.5) *p* = 0.76	−0.4 (−1.7, 2.4) *p* = 0.92
Sleep impairment	49.1 (40.2, 56.6)	50.8 (39.9, 55.5)	48.8 (40.6, 52.9)	0.7 (−2.1, 4.9) *p* = 0.46	−0.2 (−6.0, 5.1) *p* = 0.71
Mental					
Anxiety	49.3 (37.1, 53.5)	50.9 (37.1, 56.5)	47.8 (38.3, 56.3)	(−1.2, 3.0) *p* = 0.41	0.0 (−3.6, 3.0) *p* = 0.85
Depression	47.2 (38.2, 54.2)	44.5 (38.2, 53.4)	49.9 (38.2, 54.2)	0.0 (−1.5, 3.6) *p* = 0.50	0.0 (−1.7, 4.8) *p* = 0.32
Social					
Satisfaction with social roles and activities ^c^	50.5 (45.3, 58.0)	51.7 (42.7, 58.0)	51.3 (46.6, 65.4)	−1.6 (−5.6, 6.0) *p* = 0.77	3.3 (−1.4, 10.9) *p* = 0.14

^a^ *p*-values for the change scores are from the Wilcoxon Signed-Rank Test. ^b^ There was no vigorous-intensity stepping cadence according to the activPAL monitor. ^c^ Higher scores indicate better functioning for physical function and social functioning (i.e., satisfaction with social roles and activities); however, for the remaining domains, higher scores indicate worse functioning.

## Data Availability

Aggregate data may be available for research purpose upon reasonable request to the corresponding author.

## References

[1] Miller K.D., Nogueira L., Mariotto A.B., Rowland J.H., Yabroff K.R., Alfano C.M., Jemal A., Kramer J.L., Siegel R.L. (2019). Cancer treatment and survivorship statistics, 2019. CA Cancer J. Clin..

[2] American Cancer Society (2019). Cancer Treatment & Survivorship Facts & Figures 2019–2021.

[3] Avis N.E., Deimling G.T. (2008). Cancer survivorship and aging. Cancer.

[4] Hewitt M., Rowland J.H., Yancik R. (2003). Cancer survivors in the United States: Age, health, and disability. J. Gerontol. A Biol. Sci. Med. Sci..

[5] Morgans A.K., Fan K.H., Koyama T., Albertsen P.C., Goodman M., Hamilton A.S., Hoffman R.M., Stanford J.L., Stroup A.M., Resnick M.J. (2015). Influence of age on incident diabetes and cardiovascular disease in prostate cancer survivors receiving androgen deprivation therapy. J. Urol..

[6] Singh S., Earle C.C., Bae S.J., Fischer H.D., Yun L., Austin P.C., Rochon P.A., Anderson G.M., Lipscombe L. (2016). Incidence of Diabetes in Colorectal Cancer Survivors. J. Natl. Cancer Inst..

[7] Armenian S.H., Xu L., Ky B., Sun C., Farol L.T., Pal S.K., Douglas P.S., Bhatia S., Chao C. (2016). Cardiovascular Disease Among Survivors of Adult-Onset Cancer: A Community-Based Retrospective Cohort Study. J. Clin. Oncol..

[8] Strongman H., Gadd S., Matthews A., Mansfield K.E., Stanway S., Lyon A.R., Dos-Santos-Silva I., Smeeth L., Bhaskaran K. (2019). Medium and long-term risks of specific cardiovascular diseases in survivors of 20 adult cancers: A population-based cohort study using multiple linked UK electronic health records databases. Lancet.

[9] Schoormans D., Vissers P.A.J., van Herk-Sukel M.P.P., Denollet J., Pedersen S.S., Dalton S.O., Rottmann N., van de Poll-Franse L. (2018). Incidence of cardiovascular disease up to 13 year after cancer diagnosis: A matched cohort study among 32,757 cancer survivors. Cancer Med..

[10] Overholser L.S., Callaway C. (2018). Preventive Health in Cancer Survivors: What Should We Be Recommending?. J. Natl. Compr. Cancer Netw..

[11] Denlinger C.S., Ligibel J.A., Are M., Baker K.S., Demark-Wahnefried W., Dizon D., Friedman D.L., Goldman M., Jones L., King A. (2014). Survivorship: Healthy lifestyles, version 2.2014. J. Natl. Compr. Cancer Netw..

[12] Cancer.Net What Is Survivorship?. https://www.cancer.net/survivorship/what-survivorship#:~:text=A%20person%20who%20has%20had,reasons%20for%20this%20may%20vary.

[13] Jacob M.E., Yee L.M., Diehr P.H., Arnold A.M., Thielke S.M., Chaves P.H., Gobbo L.D., Hirsch C., Siscovick D., Newman A.B. (2016). Can a Healthy Lifestyle Compress the Disabled Period in Older Adults?. J. Am. Geriatr. Soc..

[14] Li Y., Pan A., Wang D.D., Liu X., Dhana K., Franco O.H., Kaptoge S., Di Angelantonio E., Stampfer M., Willett W.C. (2018). Impact of Healthy Lifestyle Factors on Life Expectancies in the US Population. Circulation.

[15] McCullough M.L., Patel A.V., Kushi L.H., Patel R., Willett W.C., Doyle C., Thun M.J., Gapstur S.M. (2011). Following cancer prevention guidelines reduces risk of cancer, cardiovascular disease, and all-cause mortality. Cancer Epidemiol. Biomarkers Prev..

[16] Heath G.W., Parra D.C., Sarmiento O.L., Andersen L.B., Owen N., Goenka S., Montes F., Brownson R.C., Lancet Physical Activity Series Working G. (2012). Evidence-based intervention in physical activity: Lessons from around the world. Lancet.

[17] Morey M.C., Snyder D.C., Sloane R., Cohen H.J., Peterson B., Hartman T.J., Miller P., Mitchell D.C., Demark-Wahnefried W. (2009). Effects of home-based diet and exercise on functional outcomes among older, overweight long-term cancer survivors: RENEW: A randomized controlled trial. JAMA.

[18] Demark-Wahnefried W., Rogers L.Q., Alfano C.M., Thomson C.A., Courneya K.S., Meyerhardt J.A., Stout N.L., Kvale E., Ganzer H., Ligibel J.A. (2015). Practical clinical interventions for diet, physical activity, and weight control in cancer survivors. CA Cancer J. Clin..

[19] Basen-Engquist K., Alfano C.M., Maitin-Shepard M., Thomson C.A., Stein K., Syrjala K.L., Fallon E., Pinto B.M., Schmitz K.H., Zucker D.S. (2018). Moving Research into Practice: Physical Activity, Nutrition, and Weight Management for Cancer Patients and Survivors. NAM Perspectives.

[20] Reis R.S., Salvo D., Ogilvie D., Lambert E.V., Goenka S., Brownson R.C., Lancet Physical Activity Series 2 Executive C. (2016). Scaling up physical activity interventions worldwide: Stepping up to larger and smarter approaches to get people moving. Lancet.

[21] USDA National Institute of Food and Agriculture: Cooperative Extension System. https://nifa.usda.gov/cooperative-extension-system.

[22] American Horticultural Society Master Gardeners. https://ahsgardening.org/gardening-resources/master-gardeners.

[23] USDA National Institute of Food and Agriculture: Horticulture Programs. https://nifa.usda.gov/program/horticulture-programs.

[24] Blair C.K., Madan-Swain A., Locher J.L., Desmond R.A., de Los Santos J., Affuso O., Glover T., Smith K., Carley J., Lipsitz M. (2013). Harvest for health gardening intervention feasibility study in cancer survivors. Acta Oncol..

[25] Bail J.R., Fruge A.D., Cases M.G., De Los Santos J.F., Locher J.L., Smith K.P., Cantor A.B., Cohen H.J., Demark-Wahnefried W. (2018). A home-based mentored vegetable gardening intervention demonstrates feasibility and improvements in physical activity and performance among breast cancer survivors. Cancer.

[26] Demark-Wahnefried W., Cases M.G., Cantor A.B., Fruge A.D., Smith K.P., Locher J., Cohen H.J., Tsuruta Y., Daniel M., Kala R. (2018). Pilot Randomized Controlled Trial of a Home Vegetable Gardening Intervention among Older Cancer Survivors Shows Feasibility, Satisfaction, and Promise in Improving Vegetable and Fruit Consumption, Reassurance of Worth, and the Trajectory of Central Adiposity. J. Acad. Nutr. Diet.

[27] Blair C.K., Harding E.M., Adsul P., Moran S., Guest D., Clough K., Sussman A.L., Duff D., Cook L.S., Rodman J. (2021). Southwest Harvest for Health: Adapting a mentored vegetable gardening intervention for cancer survivors in the southwest. Contemp. Clin. Trials Commun..

[28] Cases M.G., Fruge A.D., De Los Santos J.F., Locher J.L., Cantor A.B., Smith K.P., Glover T.A., Cohen H.J., Daniel M., Morrow C.D. (2016). Detailed methods of two home-based vegetable gardening intervention trials to improve diet, physical activity, and quality of life in two different populations of cancer survivors. Contemp. Clin. Trials.

[29] New Mexico State University College of Agricultural, Consumer, and Environmental Sciences: Cooperative Extension Service. https://extension.nmsu.edu/index.html.

[30] New Mexico State University Extension & Outreach. https://www.nmsu.edu/extension_and_outreach/.

[31] New Mexico State University College of Agricultural, Consumer and Environmental Sciences (ACS): Extension Master Gardener Program. https://aces.nmsu.edu/ces/mastergardeners/about-us.html.

[32] National Cancer Institute Fruit & vegetable screeners in the Eating at America’s Table Study (EATS). https://epi.grants.cancer.gov/diet/screeners/fruitveg/.

[33] National Cancer Institute Scoring the All-Day Screener. https://epi.grants.cancer.gov/diet/screeners/fruitveg/scoring/allday.html.

[34] Chastin S.F., Granat M.H. (2010). Methods for objective measure, quantification and analysis of sedentary behaviour and inactivity. Gait Posture.

[35] Grant P.M., Ryan C.G., Tigbe W.W., Granat M.H. (2006). The validation of a novel activity monitor in the measurement of posture and motion during everyday activities. Br. J. Sports Med..

[36] Kozey-Keadle S., Libertine A., Lyden K., Staudenmayer J., Freedson P.S. (2011). Validation of wearable monitors for assessing sedentary behavior. Med. Sci. Sports Exerc..

[37] Sellers C., Dall P., Grant M., Stansfield B. (2016). Validity and reliability of the activPAL3 for measuring posture and stepping in adults and young people. Gait Posture.

[38] Amireault S., Godin G. (2015). The Godin-Shephard leisure-time physical activity questionnaire: Validity evidence supporting its use for classifying healthy adults into active and insufficiently active categories. Percept. Mot. Skills.

[39] Amireault S., Godin G., Lacombe J., Sabiston C.M. (2015). Validation of the Godin-Shephard Leisure-Time Physical Activity Questionnaire classification coding system using accelerometer assessment among breast cancer survivors. J. Cancer Surviv..

[40] Rosenberg D.E., Norman G.J., Wagner N., Patrick K., Calfas K.J., Sallis J.F. (2010). Reliability and validity of the Sedentary Behavior Questionnaire (SBQ) for adults. J. Phys. Act. Health.

[41] PROMIS: Patient-Reported Outcomes Measurement Information System. http://www.healthmeasures.net/explore-measurement-systems/promis/intro-to-promis.

[42] Cella D., Choi S.W., Condon D.M., Schalet B., Hays R.D., Rothrock N.E., Yount S., Cook K.F., Gershon R.C., Amtmann D. (2019). PROMIS((R)) Adult Health Profiles: Efficient Short-Form Measures of Seven Health Domains. Value Health.

[43] Cook K.F., Jensen S.E., Schalet B.D., Beaumont J.L., Amtmann D., Czajkowski S., Dewalt D.A., Fries J.F., Pilkonis P.A., Reeve B.B. (2016). PROMIS measures of pain, fatigue, negative affect, physical function, and social function demonstrate clinical validity across a range of chronic conditions. J. Clin. Epidemiol..

[44] Schalet B.D., Hays R.D., Jensen S.E., Beaumont J.L., Fries J.F., Cella D. (2016). Validity of PROMIS physical function measures in diverse clinical samples. J. Clin. Epidemiol..

[45] Schalet B.D., Pilkonis P.A., Yu L., Dodds N., Johnston K.L., Yount S., Riley W., Cella D. (2016). Clinical validity of PROMIS Depression, Anxiety, and Anger across diverse clinical samples. J Clin Epidemiol..

[46] Cutrona C.E., Russell D., Jones W.H., Perlman D. (1987). The provisions of social relationships and adaptation stress. Advances in Personal Relationships.

[47] Blair C.K., Harding E., Wiggins C., Kang H., Schwartz M., Tarnower A., Du R., Kinney A.Y. (2021). A Home-Based Mobile Health Intervention to Replace Sedentary Time with Light Physical Activity in Older Cancer Survivors: Randomized Controlled Pilot Trial. JMIR Cancer.

[48] Campbell M.K., McLerran D., Turner-McGrievy G., Feng Z., Havas S., Sorensen G., Buller D., Beresford S.A., Nebeling L. (2008). Mediation of adult fruit and vegetable consumption in the National 5 A Day for Better Health community studies. Ann. Behav. Med..

[49] Stockwell S., Trott M., Tully M., Shin J., Barnett Y., Butler L., McDermott D., Schuch F., Smith L. (2021). Changes in physical activity and sedentary behaviours from before to during the COVID-19 pandemic lockdown: A systematic review. BMJ Open Sport Exerc. Med..

[50] Grajek M., Dzialach E., Buczkowska M., Gorski M., Nowara E. (2021). Feelings Related to the COVID-19 Pandemic Among Patients Treated in the Oncology Clinics (Poland). Front. Psychol..

[51] Cox B.D., Whichelow M.J., Prevost A.T. (2000). Seasonal consumption of salad vegetables and fresh fruit in relation to the development of cardiovascular disease and cancer. Public Health Nutr..

[52] Locke E., Coronado G.D., Thompson B., Kuniyuki A. (2009). Seasonal variation in fruit and vegetable consumption in a rural agricultural community. J. Am. Diet Assoc..

[53] Cepeda M., Koolhaas C.M., van Rooij F.J.A., Tiemeier H., Guxens M., Franco O.H., Schoufour J.D. (2018). Seasonality of physical activity, sedentary behavior, and sleep in a middle-aged and elderly population: The Rotterdam study. Maturitas.

[54] Tucker P., Gilliland J. (2007). The effect of season and weather on physical activity: A systematic review. Public Health.

[55] Turrisi T.B., Bittel K.M., West A.B., Hojjatinia S., Hojjatinia S., Mama S.K., Lagoa C.M., Conroy D.E. (2021). Seasons, weather, and device-measured movement behaviors: A scoping review from 2006 to 2020. Int. J. Behav. Nutr. Phys. Act..

